# Prognostic value of preoperative albumin-to-alkaline phosphatase ratio in patients with surgically treated urological cancer: a systematic review and meta-analysis

**DOI:** 10.3389/fonc.2023.1236167

**Published:** 2023-11-09

**Authors:** Shangqing Ren, Han Wang, Bo Yang, Yang Zheng, Yong Ou, Yige Bao, Yu Mao, Yunlin Feng

**Affiliations:** ^1^ Robotic Minimally Invasive Surgery, Sichuan Academy of Medical Sciences and Sichuan Provincial People’s Hospital, Chengdu, China; ^2^ Department of Gastroenterology, Sichuan Academy of Medical Sciences & Sichuan Provincial People’s Hospital, Chengdu, China; ^3^ Department of Pediatric Surgery, Sichuan Academy of Medical Sciences & Sichuan Provincial People’s Hospital, Chengdu, China; ^4^ School of Medicine, University of Electronic Science and Technology of China, Chengdu, China; ^5^ Department of Urology, Institute of Urology, West China Hospital, Sichuan University, Chengdu, China; ^6^ Department of Nephrology, Sichuan Provincial People’s Hospital, University of Electronic Science and Technology of China, Chengdu, China

**Keywords:** albumin-to-alkaline phosphatase ratio, urological cancer, prognostic value, meta-analysis, surgical

## Abstract

**Objective:**

A novel albumin-to-alkaline phosphatase ratio (AAPR) is associated with the prognosis of several cancers. In the present study, we evaluate the prognostic significance of perioperative AAPR in urological cancers.

**Method:**

Relevant studies were searched comprehensively from CNKI, PubMed, Embase and Web of Science up to March 2023. The pooled hazard ratio (HR) and 95% confidence interval (CI) were extracted from each study to evaluate the prognostic value of perioperative AAPR in patients with surgically treated urological cancers.

**Results:**

A total of 8 studies consisting of 3,271 patients were included in the final results. A low AAPR was significantly associated with a worse OS (HR=2.21; P<0.001), CSS (HR=2.61; P<0.001) and RFS/DFS (HR=2.87; P=0.001). Stratified by disease, a low AAPR was also associated with worse OS in renal cell carcinoma (HR=2.01; P<0.001), bladder cancer (HR=3.37; P<0.001) and upper tract urothelial carcinoma (HR=1.59; P=0.002).

**Conclusion:**

In conclusion, low AAPR could serve as an unfavorable factor in patients with surgically treated urological cancers. Stratified by tumor type, the low AAPR was also associated with inferior survival. While more prospective and large-scale studies are warranted to validate our findings.

## Introduction

Urological cancers, mainly consisting of renal cell carcinoma (RCC), bladder cancer (BC), and prostate cancer (PCa), represent an increased global burden on human healthcare (1). RCC accounts for approximately 2%-3% of all malignancies (1). PCa is one of the common cancers in men, ranking the second most common cancer in 2020 worldwide (2). BC is also one of the most common malignancies, with an estimated 570,000 new cases and 210,000 deaths in 2020 worldwide (2).

Although the development of novel therapeutics such as immunotherapy and molecular target drugs has greatly improved clinical outcomes of patients with urological cancers (3–5), the cornerstone of treatment for localized for urological cancers has always been surgical resection (1). The management of urological cancer still face the dilemma of low objective response rate, local recurrence, and distant metastases. Therefore, identifying the prognostic factors of patients would be of great value to patients’ risk stratification, treatment selection, and long-term outcomes prediction.

TNM stage, tumor grade, and histology are commonly used prognostic factors, yet bear the risk of missing information associated with patient-related factors. Increasing evidence has suggested that host nutrition status plays an important role in cancer development and progression (6–8), such as controlling nutritional status (CONUT) score and prognostic nutrition index (PNI) which have been found relevant to the prognosis of urological cancers (9, 10). Albumin-to-alkaline phosphatase ratio (AAPR) is another novel serum biomarker of nutritional status that has been demonstrated to be associated with the prognosis of several cancers, including lung cancer, hepatocellular carcinoma, and urological cancers (11–14); however, its role in urological cancers has only been reported in sporadic reports. There is a lack of evidence-based conclusion on the value of AAPR in urological cancer.

Therefore, we conducted this systematic review and meta-analysis to summarize all relevant studies and evaluate the prognostic significance of preoperative AAPR in patients with surgically treated urological cancers, in the hope of clarifying the value of AAPR in this field and provide evidence-based information for future studies.

## Materials and methods

### Search strategy

The study was carried out according to the Preferred Reporting Items for Systematic Reviews and Meta-Analyses (PRISMA) Statement (15). Relevant studies were searched comprehensively from CNKI, PubMed, Embase, and Web of Science up to 2023 March 08. The study search was conducted independently by two authors (SQR and HW) using search terms relevant to AAPR and urological cancers. The detailed search strategies are shown in **Supplementary Table 1**. References of eligible studies were also manually screened to avoid any omission.

### Study screening

Studies eventually included in the systematic review must meet the following criteria (1): population-based studies; (2) reported patients with surgically treated urological cancers; (3) had AAPR with accurate definition and calculation based on accepted formula; (4) evaluated the prognostic value of preoperative AAPR; (5) reported analyzable data such as hazard ratio (HR) and 95% confidence interval (CI).

Studies were excluded if they met any of the following criterion: (1) did not report AAPR; (2) did not report sufficient data for meta-analysis; (3): review and conference abstracts. For reports of the same cohort, the study with the largest and latest data was included.

The study screening was conducted independently by two authors (SQR and HW). Any discrepancy was resolved by a third author (YLF).

### Studied outcomes

The primary outcome of this systematic review is patient survival which might be reported in different modes, including overall survival (OS), cancer-specific survival (CSS), disease-free survival (DFS), and recurrence-free survival (RFS).

### Data extraction and quality assessment

Two authors (SQR and YLF) extracted the following information from eligible studies independently based on the predefined items: the surname of the first author, publication year, participant, study design, disease, interventions, number and ages of patients, the cut-off value of AAPR, and duration of follow-up. The quality of studies was assessed by the Newcastle-Ottawa Quality Assessment Scale (NOS) which includes three main aspects, namely selection, comparability, and exposure/outcome. The total NOS scores ranges from 0 to 9, and a score of 7 or higher is deemed to be high quality (16).

### Statistical analysis

All statistical analyses were performed with STATA (version 12, StataCorp, College Station, TX, USA). Pooled HRs and 95%CIs were extracted from each study to evaluate the prognostic value of AAPR in patients with urological cancers. The Cochran’s Q test and the Higgins’ I^2^ statistic were used to evaluate the heterogeneity across studies (17). If the I^2^≥50% or P<0.10, the random-effect model was used, otherwise the fixed-effect model was applied. The sensitivity analyses were performed to identify the stability of the final results by omitting each study in sequence. The publication bias was tested by Egger’s test and Begg’s test. If publication bias was detected, the trim and fill method was conducted to estimate the missing studies and recalculate the pooled HRs (18). A two-sided P-value of <0.05 was considered significant.

## Results

**Figure 1:**
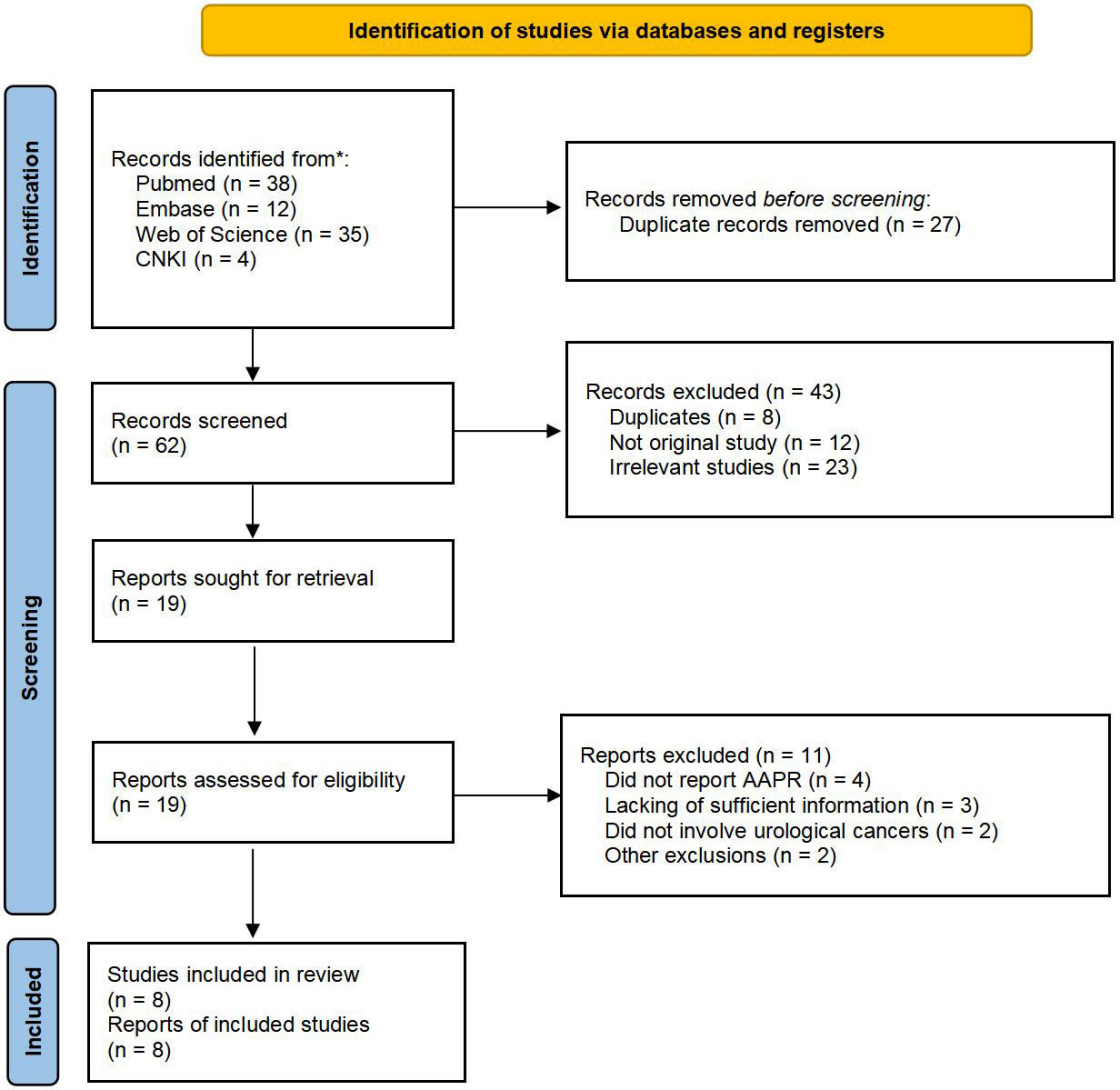
PRISMA flow diagram of the study.

A total of 89 records were identified through the electronic database search. After removing the 27 duplicated records, the remaining 62 records were screened. 19 studies were reviewed in full-text after screening based on titles and abstracts. Finally, 8 studies consisting of 3,271 patients were included in the meta-analysis (11, 13, 19–24). The detailed flow diagram was illustrated in **Figure 1**.

### Clinical characteristics of the included studies

All eight studies were retrospective cohort studies and had been published within the past 5 years. These studies reported a variety of urological cancers, including 4 for non-metastatic renal cell carcinoma (RCC) treated with nephrectomy (13, 19, 22, 23), 2 studies for BC treated with radical cystectomy (11, 24), 1 for upper tract urothelial carcinoma (UTUC) treated with radical nephroureterectomy (21), and 1 study involved prostate cancer treated with radical prostatectomy (20). The sample size of the studies ranged from 127 to 803. The cut-off value of APRP in each study is not uniform, ranging from 0.37 to 0.64. 6 studies had reported the overall survival (OS) (11, 19, 21–24), 6 studies had reported the cancer-specific survival (CSS) (11, 13, 21–24), and 5 studies reported disease-free survival (DFS)/recurrence-free survival (RFS) (11, 13, 20, 21, 24). All studies were regarded as high quality with NOS scores higher than 7. The basic characteristic of included studies were summarized in **Table 1**.

**Table 1:** Basic characteristics of included studies.

Study/Year	Study period/Location	Disease	Treatment	Number of patients	Age (years)^†^	Cut off of AAPR	Reported outcomes	Follow-up (months)^*^	NOS score
Tan 2018 (21)	2003 to 2016/China	UTUC	RUN	692	65.8 ± 11.4	0.58	OS, CSS, DFS	42 (20–75)	8
Xia 2019 (22)	January 2004 to July 2014/China	Non-metastatic RCC	Nephrectomy	803	61.0 ± 12.9	0.39	OS, CSS	50.0 (30.4-83.0)	8
Hu 2020 (23)	January 2010 to December 2013/China	Non-metastatic RCC	Nephrectomy	648	54.84 ± 12.64	0.5	OS, CSS	84	8
Zhao 2020 (24)	2007 to 2015/China	Bladder cancer	Radical cystectomy	174	Not reported	Trichotomous	OS, CSS, RFS	Median (range): 30 (1–125)	7
Li 2021 (11)	January 2012 to December 2017/China	Bladder cancer	Radical cystectomy	199	64.0 ± 8.7	Trichotomous 0.37 0.59	OS, CSS, RFS	Mean 24	7
Chen 2021 (19)	January 2012 to January 2015/China	Non-metastatic RCC	Nephrectomy	127	56.24 ± 10.13	0.4	OS	Not reported	7
Zhang 2021 (20)	May 2012 to October 2015 China	Prostate cancer	Radical prostatectomy	137	56.4 ± 20.71	Trichotomous 0.50 0.64	biochemical recurrence- free survival	55	7
Won 2022 (13)	June 1994 to December 2018/Korea	Non-metastatic RCC	Nephrectomy	491	Median (range): 56.2 (18-83)	0.41	CSS, RFS	Median (range): 63 (4-272)	8

^*^The values are median (IQR) unless specified.

^†^The values are mean ± SD unless specified.

CSS, Cancer-specific survival; DFS, Disease-free survival; IQR, Interquartile range; NOS, Newcastle-Ottawa Quality Assessment Scale; OS, Overall survival; RCC, Renal cell carcinoma; RFS, Recurrence-free survival; RUN, Radical nephroureterectomy; SD, Standard deviation; UTUC, Upper tract urothelial carcinoma.

### Survival outcomes

**Figure 2:**
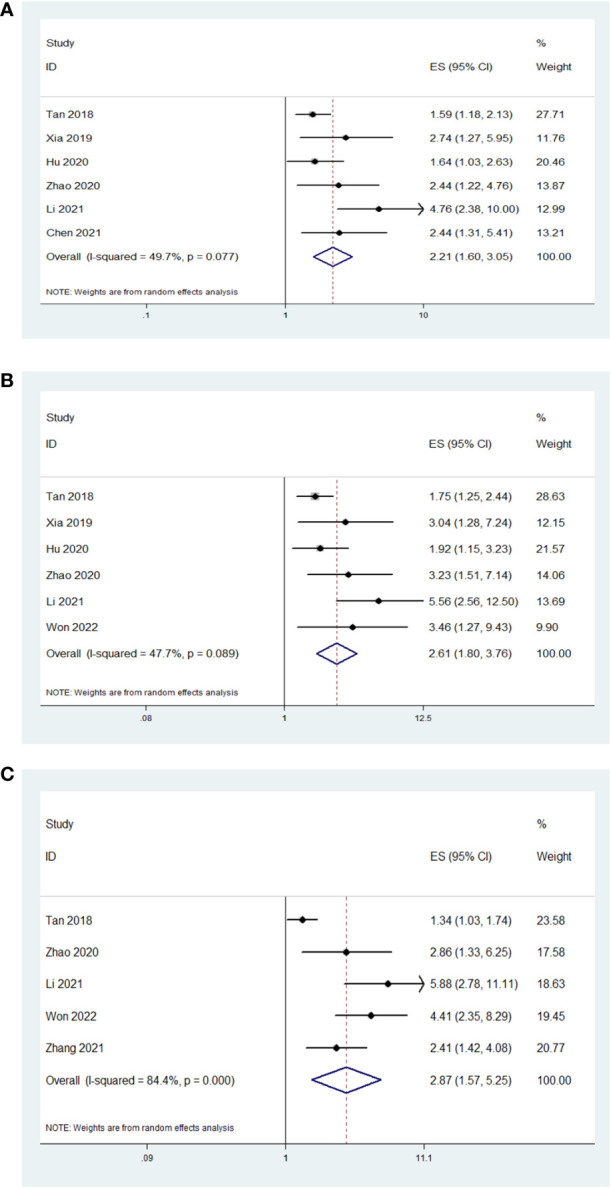
The association between AAPR and studied survival outcomes in patients with surgically treated urological cancers. Low AAPR was significantly associated with worse OS **(A)**, CSS **(B)** and DFS/RFS **(C)** compared with high AAPR.

In the six studies that had reported the OS, low AAPR was significantly associated with worse OS compared with high AAPR (HR=2.21, 95% CI 1.60-3.05, P<0.001; I^2 = ^49.7%, P=0.077; **Figure 2A**). In the six studies that had reported the CSS, low AAPR was also significantly associated with worse CSS compared with high AAPR (HR=2.61, 95% CI 1.80-3.76, P<0.001; I^2 = ^47.7%, P=0.089; **Figure 2B**). In the five studies that had reported the DFS/RFS, low AAPR again was associated with worse DFS/RFS compared with high AAPR (HR=2.87, 95% CI 1.57-5.25, P=0.001; I^2 = ^84.4%, P<0.001; **Figure 2C**).

### Sensitivity analysis

**Figure 3:**
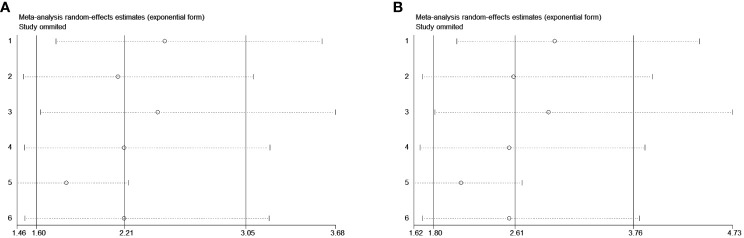
Sensitivity analysis for the studied outcomes. Removing any single study did not dramatically change the trend of our results in OS **(A)** and CSS **(B)**.

The sensitivity analysis for OS and CSS by removing each study in sequence to reflect the impact of any individual study on the overall effect indicated that removing any single study did not dramatically change the trend of our results (**Figure 3**), indicating the robustness of the results.

### Publication bias

**Figure 4:**
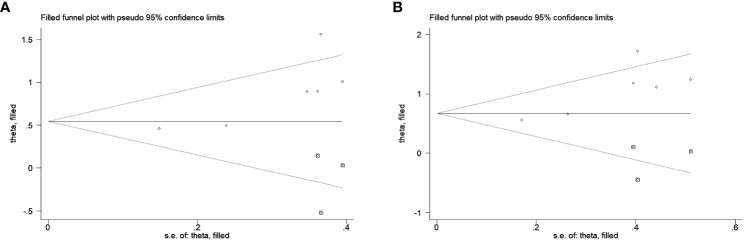
The trim and fill method to estimate publication bias for the survival outcomes. Three studies were potentially missing in OS **(A)** and CSS **(B)** using the random-effect model.

Only the publication bias for OS and CSS were evaluated due to the small number of enrolled studies. A conflicting result according to the Egger’s test (OS: P=0.038; CSS: P=0.026) and Begg’s test (OS: P=0.060; CSS: P=0.260). Therefore, we conducted the trim and fill method to identify the effect of publication bias, finding that 3 studies were potentially missing in OS and CSS using the random-effect model (**Figure 4**). This approach resulted in a similar result, the pooled HRs for OS and CSS were 1.73 (95%CI 1.23-2.43, P=0.002) and 1.96 (95%CI 1.33-2.89, P=0.001), respectively.

### Subgroup analysis

Subgroup analysis stratified by disease, number of patients and cut-off value of AAPR were conducted and indicated that disease and cut-off value of AAPR might be the source of heterogeneity of OS, but not for the CSS. In the subgroup analysis of disease, low AAPR predicts poor OS and CSS in RCC, BC, and UTUC. And in the subgroup with different sample sizes, low AAPR was also associated with the worse OS and CSS. As for cut-off value, low AAPR was an unfavorable factor in both subgroups. The detailed information was summarized in **Table 2**.

**Table 2:** Subgroup analyses of studied outcomes.

Outcome	Variable		Number of studies	HR (95% CI)	I^2^	P value of heterogeneity
OS	All		6	2.21 (1.60-3.05)	49.7%	0.077
	Disease	RCC	3	2.01 (1.42-2.84)	0	0.439
		BC	2	3.37 (1.75-6.50)	43.1%	0.185
		UTUC	1	1.59 (1.19-2.13)	–	–
	Cut-off	Dichotomous	4	1.75 (1.40-2.19)	0	0.447
		Trichotomous	2	3.37 (1.75-6.50)	43.1%	0.185
	Sample size	<200	3	3.03 (1.97-4.66)	0	0.427
		>200	3	1.68 (1.33-2.13)	11.8%	0.322
CSS	All		6	2.61 (1.80-3.76)	49.7%	0.077
	Disease	RCC	3	2.34 (1.56-3.51)	0	0.474
		BC	2	4.21 (2.42-7.33)	0	0.336
		UTUC	1	1.75 (1.25-2.44)	–	–
	Cut-off	Dichotomous	4	1.97 (1.52-2.55)	0	0.443
		Trichotomous	2	4.21 (2.42-7.33)	0	0.336
	Sample size	<200	2	4.21 (2.42-7.33)	0	0.336
		>200	4	1.97 (1.52-2.55)	0	0.443

BC, bladder cancer; CI, confidence interval; CSS, Cancer-specific survival; HR, hazards ratio; OS, overall survival; RCC, Renal cell carcinoma; UTUC, Upper tract urothelial carcinoma.

## Discussion

The present study evaluated the association between AAPR and survival outcomes of urological cancers. The findings indicated that low AAPR was associated with poor survival outcomes of urological cancers. When stratified by diseases, low AAPR also predicted worse OS and CSS in RCC, BC, and UTUC. The cut-off values of AAPR and sample sizes in each individual study varied greatly. Corresponding subgroup analysis found these factors did not significantly affect the final results.

Urological cancers account for a relatively large proportion of all solid tumors, in which local recurrence or metastasis are highly likely to occur. For example, about three-fourths of high-risk bladder cancer will recur, progress, or die within 10 years after initial diagnosis (25). Besides, nearly 30% of RCC patients will develop local or distant recurrence after surgical resection (26). The prognosis of metastatic prostate cancer is also poor, with an approximate 5-year survival rate of 30% (27). Therefore, exploring prognostic factors of urological cancers has important role in the management of this population.

The association between nutrition and malignancy has been widely explored in the past decades. Sarcopenia, the degenerative and systemic loss of skeletal muscle mass, indicates patient frailty and unfavorable prognosis in urological cancer patients (28). The prognostic nutritional index (PNI), reflecting immune and nutritional status based on the serum lymphocyte count and albumin level, is associated with prognosis of RCC (10). AAPR, a novel nutritional index, was firstly introduced and observed to be associated with the prognosis in hepatocellular carcinoma (29). Hu et al. investigated patients with surgically treated non-metastatic renal cell carcinoma and found that low AAPR was an unfavorable prognostic factor. They also found that AAPR improved the predictive value of well-established models (23). Won et al. validated the prognostic value of AAPR in patients with RCC treated with nephrectomy using propensity score matching analysis (13). Yoshino et al. demonstrated that baseline AAPR was significantly associated with OS in patients with mRCC receiving nivolumab monotherapy (30). Furthermore, AAPR could predict survival outcomes in UTUC and bladder cancer patients treated with surgery (11, 21). As for prostate cancer, Zhang et al. revealed that AAPR was associated with biochemical recurrence-free survival (20). Based on the above-mentioned evidence, high AAPR could be served as an unfavorable factor in cancer patients. However, for the other urological cancers such as testicular and penile cancer, there is no relevant report about the association between perioperative AAPR and patients’ prognosis. And more large-scale studies are required to verify our findings.

AAPR is a ready to use index in clinical practice. It is calculated based on the albumin and ALP values, which are convenient, easily obtained and commonly tested before treatment. AAPR could predict the prognosis of patients, which could be used for risk stratification. It could provide physicians with useful information and guide the treatment, adjuvant therapy, and follow-up for patients. While, the optimal cut-off value of AAPR remains unclear, which is need further exploration. The potential mechanisms for the prognostic value of AAPR might be explained by the functions of albumin and alkaline phosphatase (ALP). Albumin is a stable and abundant serum protein, reflecting the nutritional status. It also represents systemic inflammatory response, as inflammation that could influence the synthesis of albumin (31). Albumin also can stabilize cell proliferation and growth, as well as exert antioxidants agents against carcinogens (32). Evidence has found that albumin could predict prognosis in various malignancies such as RCC and bladder cancer (33, 34). ALP is a hydrolytic enzyme, found primarily in the bile duct, liver, kidney, bone, and several other organs. ALP can be affected by liver function damage from chronic wasting diseases and the cancer-related inflammatory microenvironment (35). The level of ALP level increases under certain pathological conditions, such as hepatocellular carcinoma, kidney, and bone diseases (21). ALP could also act as a potential indicator of oxidative stress and promotes high mutagenic metabolic activities, resulting in more aggressive carcinogenesis (36, 37). ALP has been reported to be associated with prognosis of gastric cancer, RCC, and hepatocellular carcinoma (38–40). Therefore, low AAPR may indicate low albumin and high ALP levels, suggesting weak nutrition and abnormal immune response in patients and finally facilitating the tumor invasion and metastasis.

Our study had limitations. First, only 8 studies consisting of 3,271 patients were included and the number of studies for each cancer is also limited, which may limit the power of final results. Second, all studies were retrospective studies with the potential inherent bias, which might account for the observed heterogeneities. Third, there are several factors unavailable including age and stage at subgroup analyses, precluding from additional investigations.

## Conclusion

In conclusion, low AAPR could serve as an unfavorable factor for the survival outcomes in patients with surgically treated urological cancers. Stratified by tumor type, the low AAPR was also associated with worse survival. More prospective and large-scale studies are warranted to validate our findings.

## Data availability statement

The original contributions presented in the study are included in the article/**Supplementary Material**. Further inquiries can be directed to the corresponding authors.

## Author contributions

SR and YF: Study conception and design, acquisition of data, analysis and interpretation of data and drafting of paper. SR, HW and YM: Acquisition of data, analysis and interpretation of data. YB, SR, and YF: Acquisition of data, drafting of paper and critical revision. YZ, YO, SR, and BY: Study conception and design, drafting of paper and critical revision. All authors contributed to the article and approved the submitted version.
