# Supplementary material for: Prognostic value of preoperative albumin-to-alkaline phosphatase ratio in patients with surgically treated urological cancer: a systematic review and meta-analysis
Source: Front Oncol. 2023 Nov 9;13:1236167. doi: 10.3389/fonc.2023.1236167 (PMC10666183; doi:10.3389/fonc.2023.1236167)
Supplement: Supplementary file 1 [file Table_1.docx]

Supplementary Table 1. Search terms.

| Data Base | Detailed search terms |
| --- | --- |
| Web of Science | ((albumin-to-alkaline phosphatase ratio) OR (albumin to alkaline phosphatase ratio) OR (Albumin/alkaline Phosphatase Ratio) OR AAPR) AND ((renal cancer) OR (renal cell carcinoma) OR (renal tumor) OR (bladder cancer) OR (bladder carcinoma) OR (bladder tumor) OR (urothelial cancer) OR (urothelial carcinoma) OR (urothelial tumor) OR (prostate cancer)) |
| Embase | 1. albumin-to-alkaline phosphatase ratio.mp.  2. albumin to alkaline phosphatase ratio.mp.  3. AAPR.mp.  4. 1 or 2 or 3  5. renal cancer.mp. or exp kidney cancer/  6. renal cell carcinoma.mp. or exp renal cell carcinoma/  7. renal tumor.mp. or exp kidney tumor/  8. 5 or 6 or 7  9. bladder cancer.mp. or exp bladder cancer/  10. bladder carcinoma.mp. or exp bladder carcinoma/  11. bladder tumor.mp. or exp bladder tumor/  12. urothelial cancer.mp. or exp transitional cell carcinoma  13. urothelial carcinoma.mp. or exp transitional cell carcinoma/  14. urothelial tumor.mp. or exp urothelial tumor/  15. 9 or 10 or 11 or 12 or 13 or 14  16. 8 or 15  17. 4 and 16  18. prostate cancer.mp. or exp prostate cancer/  19. 4 and 18 |
| Web of Science | 1. (((TS=(albumin-to-alkaline phosphatase ratio)) OR TS=(albumin to alkaline phosphatase ratio)) OR TS=(Albumin/alkaline Phosphatase Ratio)) OR TS=(AAPR)  2. ((((((((TS=(renal cancer)) OR TS=(renal cell carcinoma)) OR TS=(renal tumor)) OR TS=(bladder cancer)) OR TS=(bladder carcinoma)) OR TS=(bladder tumor)) OR TS=(urothelial cancer)) OR TS=(urothelial carcinoma)) OR TS=(urothelial tumor)  3. #1 AND #2  4. TS=(prostate cancer)  5. #1 AND #4  6. #3 OR #5 |
| CNKI | ((albumin-to-alkaline phosphatase ratio) OR (albumin to alkaline phosphatase ratio) OR (Albumin/alkaline Phosphatase Ratio) OR AAPR) AND ((renal cancer) OR (renal cell carcinoma) OR (renal tumor) OR (bladder cancer) OR (bladder carcinoma) OR (bladder tumor) OR (urothelial cancer) OR (urothelial carcinoma) OR (urothelial tumor) OR (prostate cancer)) |
